# Accelerated Biological Aging, Neurodegenerative Disease, and Mortality in Cardiovascular Disease Patients: Mediation and Modification Analysis

**DOI:** 10.1002/cns.71059

**Published:** 2026-07-29

**Authors:** Jinyue Li, Han Ma, Guohua Wang

**Affiliations:** ^1^ National Stroke Administration Office Xuanwu Hospital Capital Medical University Beijing China; ^2^ Stroke Prevention, Treatment & Translational Research Laboratory Xuanwu Hospital Capital Medical University Beijing China; ^3^ National Center for Clinical Laboratories, Beijing Hospital, National Center for Gerontology, National Clinical Research Center for Gerontology, The Key Laboratory of Geriatrics of NHC, Institute of Geriatric Medicine Chinese Academy of Medical Sciences Beijing China

**Keywords:** biological aging, cardiovascular disease patients, mortality risk, neurodegenerative diseases, secondary prevention

## Abstract

**Aims:**

Cardiovascular disease (CVD) patients exhibit increased neurodegenerative diseases and mortality risks, implying a shared heart‐brain aging pathway. As a composite biological aging predictor, the association of Phenotypic age (PhenoAge) with mortality and the mediating role of brain health remain unclear in CVD patients.

**Methods:**

In 4104 CVD patients (57.3% male, mean age 67.3 years) from the National Health and Nutrition Examination Survey (median follow‐up of 7.2 years), weighted regression models examined associations of PhenoAge and its acceleration with Alzheimer's disease (AD), Parkinson's disease (PD), and mortality. Mediation effects of the association between PhenoAge and mortality explained by AD and PD were quantified.

**Results:**

The mean PhenoAge was 69.9 years, and AD and PD prevalences were 7.6% and 1.8%. Each 5‐year increment of PhenoAge acceleration independently increased risks of AD (OR = 1.24; 95% CI:1.13,1.36), PD (OR = 1.22; 95% CI:1.02,1.46), and all‐cause mortality (HR = 1.30; 95% CI:1.24,1.36). AD and PD mediated 20.46%–33.18% of the association between PhenoAge and mortality. Early‐onset CVD amplified biological aging‐related mortality risk, while a healthier lifestyle attenuated the CVD mortality risk (*P*_interaction_ < 0.05).

**Conclusion:**

Accelerated biological aging was associated with adverse brain health outcomes and mortality in CVD patients, with AD and PD as significant mediators. PhenoAge assessment may identify high‐risk individuals for personalized heart‐brain aging prevention.

## Graphical abstract

In cardiovascular disease (CVD) patients, accelerated biological aging is independently associated with elevated mortality risk, which is mediated by neurodegenerative disease. Early‐onset CVD amplifies the mortality risk, whereas a healthy lifestyle confers partial protection. These findings highlight the heart–brain aging axis and suggest that biological aging assessment may aid in risk stratification and secondary prevention.

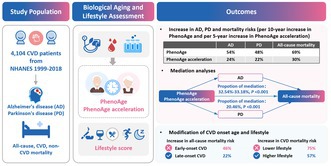

## 1 Introduction

Cardiovascular diseases (CVDs) have collectively remained the leading cause of death worldwide and imposed a substantial global health burden [1]. CVD patients not only face the risk of premature death, but also suffer from the substantial burden of cognitive dysfunction and neurodegenerative diseases (NDDs) such as Alzheimer's disease (AD) and Parkinson's disease (PD), constituting the complex clinical landscape of cardio‐cerebral comorbidity [2, 3, 4]. This phenomenon indicates the potential common pathophysiological pathways modulating the heart–brain axis [2, 5]. Existing research suggested that aging could play an important role in heart‐brain connection and disease prognosis [6, 7]. However, the risk stratification of CVD patients, which mainly relies on age and traditional risk factors, neither captures the heterogeneity of biological aging nor considers the risk of impaired brain health.

Significantly, chronological age does not adequately reflect the interindividual divergence of physiological decline and systemic dysregulation [8, 9]. Health trajectory and susceptibility to NDDs might vary considerably even in individuals of the same chronological age [10]. In recent years, composite predictors derived from routine clinical biomarkers have emerged as promising tools to quantify biological aging [11, 12]. Among these, phenotypic age (PhenoAge) is a reliable indicator trained based on a mortality prediction score [13, 14]. Nevertheless, related evidence is scarce among CVD patients, and limited studies have systematically investigated the associations of biological aging with adverse brain health and long‐term mortality risk among CVD patients.

Furthermore, as aging‐related diseases, NDDs might serve as key drivers underlying the elevated mortality linked to accelerated aging [15, 16]. Therefore, quantifying the proportion of the association between accelerated aging and mortality mediated by NDDs may provide evidence for the heart‐brain interplay aging mechanisms and help identify potential intervention targets. Besides, the above associations are likely to be influenced by several factors. The onset age of CVD might influence patient's susceptibility to biological aging and contribute to potential disparities in the health damage, whereas a healthy lifestyle may decelerate the aging process and mitigate its adverse outcomes [17, 18]. At present, the potential modifying effects of these factors have not been investigated systematically.

Hence, we aimed to evaluate the associations of PhenoAge with cognitive dysfunction, NDDs (AD and PD), and mortality, quantify the mediating effects of AD and PD on the relationship between PhenoAge and mortality, and explore potential modification effects by CVD onset age and lifestyle among CVD patients.

## 2 Methods

### 2.1 Study Design and Participants

This longitudinal study used data from the National Health and Nutrition Examination Survey (NHANES). NHANES is an ongoing national survey of the US noninstitutionalized civilian population, utilizing a complex, multistage, probability sampling design. It is conducted by the National Center for Health Statistics (NCHS), including health examinations, laboratory tests, and dietary interviews. The NCHS Ethics Review Board approved the survey protocols of NHANES and written informed consent was obtained from all participants at enrollment. Detailed information and all datasets can be found at https://www.cdc.gov/nchs/nhanes/index.html.

Data from the 1999–2018 NHANES cycles were selected for this study, with 101,316 participants at baseline. CVD was defined as a self‐reported physician diagnosis of coronary heart disease (CHD), stroke, myocardial infarction, angina pectoris, or congestive heart failure. After excluding participants younger than 18 years (*n* = 42,112), those without CVD (*n* = 52,866), those with missing PhenoAge information (*n* = 2,225), and those with missing survival information (*n* = 9), a total of 4104 participants were included in the analyses finally. Among them, 543 individuals underwent cognitive function assessment (Figure S1).

### 2.2 Laboratory Measurements

The blood biomarkers for calculating biological aging were measured from blood samples collected and processed following standardized protocols described in the NHANES Laboratory/Medical Technologists Procedures Manual, including albumin, creatinine, alkaline phosphatase (ALP), C‐reactive protein (CRP), total cholesterol (TC), lymphocyte percent (Lym%), mean cell volume (MCV), white blood cell count (WBC), glucose, and red cell distribution width (RDW). Serum and whole blood samples were analyzed at certified laboratories. Specifically, albumin, ALP, creatinine, glucose, and TC were quantified on Beckman series clinical analyzers using established bichromatic digital endpoint method, enzymatic rate method, Jaffe rate method, glucose oxidase method, and enzymatic method, respectively. High‐sensitivity CRP was determined by latex‐enhanced nephelometry. Complete blood count parameters were analyzed using a Beckman Coulter MAXM at Mobile Examination Centers. All assays adhered to rigorous quality control standards as specified by NHANES. No extreme biologically implausible outliers were detected in the datasets, and the values representing true physiological variations were retained.

### 2.3 Determination of Biological Aging Indicator

We estimated each participant's biological aging status through PhenoAge. PhenoAge is constructed using composite clinical chemistry biomarkers based on parametrization from a Gompertz mortality model and reflects the age in the general population that corresponds with an individual's mortality risk [13, 14]. The formula of PhenoAge selected nine biomarkers and chronological age.

$$\text{PhenoAge}=141.50+\frac{\ln\left[-0.00553\times \ln\left(1-\text{mortality risk}\right)\right]}{0.09165}$$

$$\text{mortality risk}=1-e^{\frac{-1.51714\times e^{\mathrm{xb}}}{0.0076927}}$$

$$\begin{array}{c} \mathrm{xb}=-19.907-0.0336\times \text{albumin}+0.0095\times \text{creatinine}\\ \;+0.1953\times \text{glucose}+0.0954\times \ln\mathrm{CRP}-0.0120\times \mathrm{Lym}\%\\ \;+0.0268\times \mathrm{MCV}+0.3306\times \mathrm{RDW}+0.00188\times \mathrm{ALP}+0.0554\times \mathrm{WBC}\\ \;+0.0804\times \text{chronological}\;\mathrm{age} \end{array}$$

The BioAge R package was applied to calculate PhenoAge (http://github.com/dayoonkwon/BioAge), which utilizes the NHANES for training and testing algorithms [19]. Afterwards, the acceleration of PhenoAge was calculated as the residuals resulting from models when regressing PhenoAge on chronological age. Individuals with positive values of PhenoAge acceleration were regarded as biologically older and those with negative values were regarded as biologically younger.

### 2.4 Assessment of Cognitive Function and Neurodegenerative Diseases

Cognitive performance was measured using the digit symbol substitution test (DSST), a subtest of the Wechsler Adult Intelligence Scale. The DSST assesses processing speed, executive function, attention, visuomotor coordination, and working memory, which were known to be sensitive to aging [20]. Participants were provided with a digit‐symbol key and asked to write the corresponding symbol under each digit as quickly as possible within 2 min. The total number of correctly matched symbols (range 0–133) served as the DSST score, with higher scores indicating better performance. A score lower than 34 indicated cognitive dysfunction. DSST is a validated measure of specific cognitive domains, and it does not constitute a comprehensive assessment of global cognitive ability or dementia status.

Based on definition used in previous studies, prevalent AD at baseline was defined based on the participants’ self‐report use of prescribed AD‐related medications (Donepezil, Galantamine, Rivastigmine, Memantine, Gabapentin) [21]. Similarly, PD was defined by self‐reported use of antiparkinsonian medications (Carbidopa, Levodopa, Ropinirole, Entacapone, Benztropine, Amantadine, Methyldopa) [22]. These definitions have high specificity because these medications are almost exclusively prescribed for AD or PD, but their sensitivity is limited as they capture only diagnosed and treated cases.

### 2.5 Ascertainment of Mortality

Mortality status and cause of death for all participants were ascertained by probabilistically linking survey records to the National Death Index death certificate database, with follow‐up through December 31, 2019. Person‐years of follow‐up were calculated from the date of the baseline examination to the date of death or the censoring date (December 31, 2019), whichever occurred first. The underlying cause of death was classified according to the International Classification of Diseases, Tenth Revision (ICD‐10). Deaths attributed to diseases of the heart (ICD‐10 codes I00‐I09, I11, I13, I20‐I51) or cerebrovascular disease (I60‐I69) were defined as CVD mortality. All other causes of death were classified as non‐CVD mortality.

### 2.6 Lifestyle and Covariate Assessment

A composite lifestyle score was derived for each participant based on five factors: smoking, alcohol consumption, physical activity, diet quality, and sleep duration. Each factor was assigned a binary score of 0 (unfavorable) or 1 (favorable). Smoking status was categorized as favorable for those who reported having smoked fewer than 100 cigarettes in their lifetime and unfavorable otherwise. For alcohol consumption, a favorable score was assigned to individuals who reported either consuming fewer than 12 alcoholic drinks in their lifetime or abstaining from alcohol in the preceding year. Physical activity was assessed using self‐reported leisure‐time activities. Weekly metabolic equivalent of task hours was calculated, and participants in the highest sex‐specific tertile were considered to have a favorable level. Diet quality was evaluated using the Dietary Approaches to Stop Hypertension (DASH) index based on a validated semiquantitative food frequency questionnaire (FFQ) consisting of over 130 questions. The DASH index includes nine dietary components (total fat, saturated fat, protein, fiber, cholesterol, calcium, magnesium, sodium, and potassium), and higher DASH scores are indicative of better diet quality [23]. A favorable diet was defined as a DASH index in the highest population tertile. Finally, sleep duration was considered favorable if self‐reported habitual sleep time was 7–9 h per night. A lifestyle score of 0 to 1 was defined as the lower lifestyle score group, while a lifestyle score of 2 to 5 was defined as the higher lifestyle score group.

Covariates were obtained from standardized questionnaires, laboratory examinations, and physical measurements conducted during the NHANES mobile examination center visit. Chronological age (years) and gender (male/female) were self‐reported. Smoking status was categorized as current smoker, former smoker, or never smoker, based on self‐reported lifetime cigarette use (≥ 100 cigarettes) and the situation of quitting smoking. Alcohol consumption status was classified as current drinker, former drinker, or never drinker, derived from detailed interview questions regarding lifetime and recent intake patterns. Education was classified into less than 9th grade or 9–11th grade, high school graduate/GED or some college/AA degree, and college graduate or above. The family poverty‐to‐income ratio (PIR) was dichotomized at a threshold of 1.0. Body mass index (BMI) was calculated as weight (kg)/height (m)^2^. Comorbidities, including hypertension, diabetes, and dyslipidemia, were defined based on a combination of self‐reported physician diagnosis, use of relevant medications, and/or established laboratory or examination criteria according to standard NHANES definitions.

### 2.7 Statistical Analyses

All analyses accounted for the complex survey design of NHANES and were performed using appropriate sample weights. The characteristics of the study participants were described stratified by PhenoAge acceleration quartiles. Continuous variables are presented as mean ± standard deviation, and categorical variables as frequency (percentage). We used weighted linear regression to test the association of PhenoAge and acceleration with DSST score, and weighted logistic regression to analyze the associations of PhenoAge and acceleration with prevalent cognitive dysfunction, AD, and PD, calculating odds ratios (ORs) and 95% confidence intervals (CIs). The associations of PhenoAge and acceleration with all‐cause, CVD, and non‐CVD mortality were evaluated using weighted Cox proportional hazards regression models, calculating hazard ratios (HRs) and 95% CIs. Weighted Kaplan–Meier curves were generated to visualize survival probabilities across quartile groups. Model 1 was a crude model, and model 2 was adjusted for age, gender, smoking, alcohol consumption, education, and family PIR. Model 3 was a full‐adjusted model, which was additionally adjusted for BMI, hypertension, diabetes, dyslipidemia, and CHD. To explore the exposure‐response relationship, we applied restricted cubic spline (RCS) models with 3 knots to examine the potential non‐linear associations of continuous PhenoAge and acceleration with all outcomes. Receiver operating characteristic (ROC) curve analysis was used to evaluate the predictive effects of PhenoAge and acceleration on cognitive dysfunction, AD, and PD, and time‐dependent ROC curve analysis was used to evaluate the predictive ability for mortality at 1, 3, 5, and 10‐year follow‐up intervals. The area under the curve (AUC) was calculated to evaluate discriminative ability.

Then, we assessed whether prevalent NDDs mediated the associations of PhenoAge and acceleration with mortality. Given that the mediator (AD or PD) was binary and the mortality was a time‐to‐event outcome, we adopted a regression‐based framework with bootstrap resampling. The indirect effect was calculated using product‐of‐coefficients approach, and the proportion mediated was computed as (indirect/total effect) × 100%. To obtain 95% CIs for the indirect effect and proportion mediated, we performed bias‐corrected bootstrap with 1000 resamples. Finally, stratified analyses were conducted to examine potential effect modification. Participants were divided into early‐onset and late‐onset CVD subgroups based on a cutoff age of 55 years for CVD onset [24], and were stratified according to lifestyle scores (lower vs. higher), hypertension, diabetes, and dyslipidemia status. In each stratum, the association between biological aging and the outcome was evaluated, and the interaction was formally tested by including multiplicative term in the model. Two sensitivity analyses were performed, including the exclusion of participants with cancer or kidney failure, as well as additional adjustment for antihypertensive and glucose‐lowering medications. A two‐sided *p* < 0.05 was considered statistically significant. All analyses were performed using R statistical software (version 4.4.3).

## 3 Results

### 3.1 Baseline Participant Characteristics

Among 4104 CVD patients, the overall mean age was 67.3 ± 13.0 years, and 57.3% were male. The mean PhenoAge was 69.9 years, and AD and PD prevalences were 7.6% and 1.8%, respectively. Stratified by quartiles of PhenoAge acceleration, participants in the higher quartiles were significantly older, more likely to be male, and had a higher BMI. Regarding lifestyle, participants with higher PhenoAge acceleration were less likely to have lower proportions of favorable smoking, physical activity, dietary, and sleep scores. The prevalence of hypertension, diabetes, AD, and PD increased markedly across quartiles (Table 1).

**TABLE 1:** Descriptive characteristics of study population by PhenoAge acceleration.

	Total (*n* = 4104)	PhenoAge acceleration quartiles				*p*
		Q1 (*n* = 1026)	Q2 (*n* = 1026)	Q3 (*n* = 1026)	Q4 (*n* = 1026)	
Age, years	67.3 ± 13.0	66.5 ± 13.4	66.3 ± 14.1	67.9 ± 12.9	68.6 ± 11.5	< 0.001
Men, *n* (%)	2350 (57.3)	480 (46.8)	597 (58.2)	631 (61.5)	642 (62.6)	< 0.001
Race, *n* (%)						< 0.001
Mexican American	497 (12.1)	172 (16.8)	114 (11.1)	103 (10.0)	108 (10.5)	
Other Hispanic	231 (5.6)	68 (6.6)	53 (5.2)	51 (5.0)	59 (5.8)	
Non‐Hispanic White	2388 (58.2)	614 (59.8)	621 (60.5)	601 (58.6)	552 (53.8)	
Non‐Hispanic Black	772 (18.8)	123 (12.0)	183 (17.8)	211 (20.6)	255 (24.9)	
Other Race	216 (5.3)	49 (4.8)	55 (5.4)	60 (5.8)	52 (5.1)	
Education, *n* (%)						0.003
Less Than 9th Grade	745 (18.2)	217 (21.2)	178 (17.3)	163 (15.9)	187 (18.2)	
9‐11th Grade	745 (18.2)	168 (16.4)	170 (16.6)	199 (19.4)	208 (20.3)	
High School Grad/GED or Equivalent	1002 (24.4)	230 (22.4)	255 (24.9)	256 (25.0)	261 (25.4)	
Some College or AA degree	1038 (25.3)	245 (23.9)	274 (26.7)	273 (26.6)	246 (24.0)	
College Graduate or above	566 (13.8)	166 (16.2)	149 (14.5)	132 (12.9)	119 (11.6)	
Family PIR < 1, *n* (%)	777 (18.9)	153 (14.9)	220 (21.4)	195 (19.0)	209 (20.4)	0.001
BMI, kg/m^2^	29.8 ± 6.7	27.8 ± 5.1	29.4 ± 6.2	30.3 ± 6.7	31.9 ± 7.8	< 0.001
High lifestyle score, *n* (%)	371 (26.4)	115 (34.1)	97 (27.1)	74 (19.9)	85 (24.9)	< 0.001
High smoking score, *n* (%)	1597 (39.0)	460 (44.8)	400 (39.0)	374 (36.5)	363 (35.4)	< 0.001
High drinking score, *n* (%)	1596 (50.0)	392 (45.2)	382 (46.5)	401 (51.1)	421 (58.4)	< 0.001
High physical activity score, *n* (%)	477 (15.8)	170 (23.0)	123 (16.5)	111 (14.6)	73 (9.5)	< 0.001
High dietary score, *n* (%)	844 (36.7)	252 (43.9)	210 (36.0)	189 (32.4)	193 (34.4)	< 0.001
High sleeping score, *n* (%)	1222 (45.8)	260 (47.6)	316 (49.5)	301 (42.3)	345 (44.6)	0.042
PhenoAge, years	69.9 ± 15.9	60.3 ± 13.6	65.1 ± 14.1	71.5 ± 13.0	82.8 ± 13.3	< 0.001
PhenoAge acceleration, years	2.6 ± 8.4	−6.2 ± 2.2	−1.2 ± 1.2	3.6 ± 1.6	14.2 ± 6.7	< 0.001
Hypertension, *n* (%)	3150 (76.8)	731 (71.2)	757 (73.8)	800 (78.0)	862 (84.0)	< 0.001
Diabetes, *n* (%)	974 (23.7)	101 (9.8)	128 (12.5)	247 (24.1)	498 (48.5)	< 0.001
Dyslipidemia, *n* (%)	2564 (62.5)	653 (63.6)	624 (60.8)	634 (61.8)	653 (63.6)	0.457
Onset age of CVD, years	55.8 ± 16.5	54.9 ± 17.2	55.3 ± 17.2	56.5 ± 15.6	56.5 ± 15.8	0.053
AD, *n* (%)	311 (7.6)	49 (4.8)	60 (5.8)	83 (8.1)	119 (11.6)	< 0.001
PD, *n* (%)	72 (1.8)	11 (1.1)	11 (1.1)	27 (2.6)	23 (2.2)	0.009
All‐cause mortality, *n* (%)	1919 (46.8)	425 (41.4)	449 (43.8)	490 (47.8)	555 (54.1)	< 0.001
CVD mortality, *n* (%)	782 (19.1)	180 (17.5)	180 (17.5)	202 (19.7)	220 (21.4)	0.069
Non‐CVD mortality, *n* (%)	1137 (27.7)	245 (23.9)	269 (26.2)	288 (28.1)	335 (32.7)	< 0.001

Abbreviations: AD, Alzheimer's disease; BMI, body mass index; CVD, cardiovascular disease; PD, Parkinson's disease; PhenoAge, phenotypic age; PIR, poverty‐to‐income ratio.

A total of 543 CVD patients with complete data on PhenoAge and cognitive function assessment were included in this analysis. The mean age of the participants was 73.3 ± 7.8 years, and 59.3% were male. Detailed baseline characteristics were presented in Table S1.

### 3.2 Associations of Biological Aging and Acceleration With Cognitive Function and Neurodegenerative Diseases

Elevated PhenoAge and PhenoAge acceleration were independently correlated with impaired cognitive function among 543 CVD patients (Table 2). In the fully adjusted model, each 5‐year increment in PhenoAge acceleration was associated with a 1.50‐point reduction in DSST score (95% CI: −2.23, −0.77) and a 24% higher risk of cognitive dysfunction prevalence (OR = 1.24; 95% CI: 1.04, 1.48). When stratified by quartiles, compared to the lowest PhenoAge acceleration quartile, participants in the top quartile exhibited markedly lower DSST performance (*β* = −5.79; 95% CI: −8.57, −3.02) and a 2.03‐fold increased cognitive dysfunction risk (95% CI: 1.10, 3.74), with significant linear trends (*P*_trend_ < 0.05). Binary comparisons further demonstrated that biologically older CVD patients had substantially worse cognitive performance than their biologically younger counterparts. Consistently, per 10‐year increase in PhenoAge was also linked to significantly decreased DSST scores (*β* = −3.00, 95% CI: −4.45, −1.54) and greater susceptibility to cognitive dysfunction (OR = 1.55, 95% CI: 1.09, 2.20).

**TABLE 2:** Association of PhenoAge and acceleration with the cognitive function among CVD patients.

	DSST score, *β* (95% CI)			Cognitive dysfunction, OR (95% CI)			
	Model 1	Model 2	Model 3	Event/N	Model 1	Model 2	Model 3
PhenoAge							
Per 10 years increase	−5.59 (−6.76, −4.42)	−2.50 (−3.76, −1.23)	−3.00 (−4.45, −1.54)	230/543	1.85 (1.56, 2.19)	1.62 (1.23, 2.14)	1.55 (1.09, 2.20)
By quartile							
Q1	0 (reference)	0 (reference)	0 (reference)	37/136	1.00 (reference)	1.00 (reference)	1.00 (reference)
Q2	−8.47 (−12.57, −4.37)	−4.52 (−7.71, −1.33)	−4.85 (−8.30, −1.39)	55/135	2.16 (1.18, 3.97)	1.63 (0.78, 3.42)	1.69 (0.77, 3.71)
Q3	−9.56 (−14.39, −4.73)	−3.26 (−8.09, 1.57)	−3.79 (−8.11, 0.52)	57/136	2.52 (1.17, 5.46)	1.46 (0.55, 3.88)	1.44 (0.57, 3.66)
Q4	−17.41 (−21.02, −13.81)	−8.65 (−12.74, −4.55)	−9.35 (−13.82, −4.87)	81/136	5.76 (3.29, 10.09)	3.01 (1.26, 7.21)	3.00 (1.10, 8.18)
*P*_trend_	< 0.001	0.001	0.001		< 0.001	0.001	0.006
PhenoAge acceleration							
Per 5 years increase	−1.96 (−2.81, −1.11)	−1.25 (−1.88, −0.62)	−1.50 (−2.23, −0.77)	230/543	1.30 (1.12, 1.50)	1.27 (1.11, 1.46)	1.24 (1.04, 1.48)
By quartile							
Q1	0 (reference)	0 (reference)	0 (reference)	46/136	1.00 (reference)	1.00 (reference)	1.00 (reference)
Q2	−3.98 (−8.17, 0.21)	−2.39 (−5.44, 0.66)	−2.66 (−5.88, 0.57)	54/135	1.41 (0.75, 2.62)	1.19 (0.63, 2.25)	1.24 (0.62, 2.46)
Q3	−5.12 (−9.24, −1.01)	−1.00 (−4.58, 2.57)	−1.11 (−4.68, 2.46)	60/136	1.79 (0.98, 3.26)	1.23 (0.63, 2.41)	1.38 (0.75, 2.52)
Q4	−7.91 (−11.45, −4.37)	−4.88 (−7.67, −2.10)	−5.79 (−8.57, −3.02)	70/136	2.44 (1.32, 4.53)	2.08 (1.20, 3.60)	2.03 (1.10, 3.74)
*P*_trend_	< 0.001	0.003	0.028		0.002	0.003	0.028
By binary							
Biologically younger	0 (reference)	0 (reference)	0 (reference)	120/323	1.00 (reference)	1.00 (reference)	1.00 (reference)
Biologically older	−5.20 (−7.98, −2.43)	−2.87 (−5.63, −0.10)	−2.84 (−5.94, 0.25)	110/220	1.78 (1.13, 2.80)	1.54 (0.89, 2.64)	1.41 (0.75, 2.66)

*Note:* The analyses for cognitive function (DSST score and cognitive dysfunction) were conducted in 543 CVD patients with complete cognitive assessment data. Model 1 was crude model; model 2 was adjusted for age, gender, smoking, alcohol consumption, education, and family PIR; model 3 was additionally adjusted for BMI, hypertension, diabetes, dyslipidemia, and CHD.

Abbreviations: BMI, body mass index; CHD, coronary heart disease; CVD, cardiovascular disease; DSST, digit symbol substitution test; OR, odds ratio; PhenoAge, phenotypic age; PIR, poverty‐to‐income ratio; 95% CI, 95% confidence interval.

**FIGURE 1:**
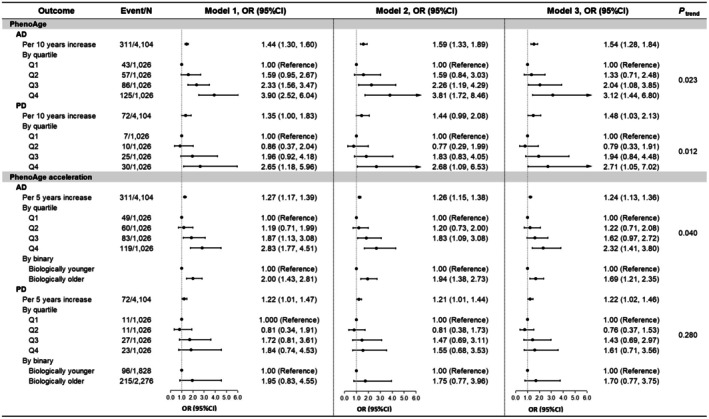
Associations of PhenoAge and acceleration with AD and PD among CVD patients. Abbreviations: AD, Alzheimer's disease; BMI, body mass index; CHD, coronary heart disease; CVD, cardiovascular disease; HR, hazard ratio; PD, Parkinson's disease; PhenoAge, phenotypic age; PIR, poverty‐to‐income ratio; 95% CI, 95% CI confidence interval. Model 1 was a crude model; model 2 was adjusted for age, gender, smoking, alcohol consumption, education, and family PIR; model 3 was additionally adjusted for BMI, hypertension, diabetes, dyslipidemia, and CHD.

In the complete analyzed dataset of 4104 CVD patients, prominent positive associations of PhenoAge and acceleration with prevalent AD and PD were observed (Figure 1). Every 5‐year increase in PhenoAge acceleration independently increased the risk of AD prevalence by 24% (OR = 1.24, 95% CI: 1.13, 1.36) and PD by 22% (OR = 1.22, 95% CI: 1.02, 1.46). CVD patients in the highest PhenoAge acceleration group had 2.32‐fold higher AD risk (95% CI: 1.41, 3.80) than those in the lowest group (*p*_trend_ = 0.040). PhenoAge was significantly associated with the prevalence of both AD and PD. For example, a 10‐year increment in PhenoAge was associated with 54% higher AD risk (OR = 1.54, 95% CI: 1.28, 1.84) and 48% higher PD risk (OR = 1.48, 95% CI: 1.03, 2.13). The RCS analysis indicated that PhenoAge was linearly and positively correlated with the risks of AD and PD, and the PhenoAge acceleration presented non‐linear exposure‐response patterns for both outcomes (Figure S2).

ROC analyses demonstrated that PhenoAge acceleration exhibited favorable predictive performance for adverse brain health outcomes in CVD patients, with an AUC of 0.787 for cognitive dysfunction and moderate significant predictive values for AD (AUC = 0.670) and PD (AUC = 0.662) (Figure S3).

### 3.3 Associations of Biological Aging and Acceleration With Mortality Risk

During 32,552 person‐years of observation (with a median follow‐up of 7.2 years), a total of 1919 deaths occurred, including 782 CVD deaths and 1137 non‐CVD deaths. Weighted Kaplan–Meier curves demonstrated significant differences in survival probabilities for all‐cause, CVD, and non‐CVD mortality across quartile strata of PhenoAge and acceleration (log‐rank *p* < 0.001) (Figure S4), showing a progressively significant downward trend in cumulative survival rate with increasing biological aging. In full‐adjusted Cox regression models, each 5‐year increase in PhenoAge acceleration was associated with a 30% (HR = 1.30; 95% CI: 1.24, 1.36), 28% (HR = 1.28; 95% CI: 1.20, 1.36), and 31% (HR = 1.31; 95% CI: 1.24, 1.38) higher risks of all‐cause, CVD, and non‐CVD mortality, respectively (Table 3). When stratified by quartiles and compared to the lowest quartile, the HRs (95% CIs) for all‐cause mortality associated with the second, third, and fourth quartiles were 1.29 (1.08, 1.54), 1.65 (1.39,1.95), and 3.11 (2.52, 3.83) (*P*_trend_ < 0.001). Similar associations of PhenoAge acceleration with CVD and non‐CVD mortality were observed. With regard to PhenoAge, greater PhenoAge remained an independent risk factor for all‐cause, CVD, and non‐CVD mortality risk (Table 3). Exposure‐response analyses showed a significant positive association between biological aging and mortality, and no significant non‐linear relationship was observed, excluding PhenoAge acceleration and non‐CVD mortality (Figure S5). Additionally, we analyzed the associations among patients with stroke or CHD, and found similar results (Tables S2, S3).

**TABLE 3:** Association of PhenoAge and acceleration with the mortality risks among CVD patients.

	Event/N	Model 1	Model 2	Model 3
PhenoAge				
All‐cause mortality				
Per 10 years increase	1919/4104	1.90 (1.80, 2.00)	1.64 (1.50, 1.80)	1.69 (1.53, 1.85)
By quartile				
Q1	191/1026	1.00 (reference)	1.00 (reference)	1.00 (reference)
Q2	384/1026	3.12 (2.60, 3.75)	1.75 (1.37, 2.24)	1.69 (1.32, 2.15)
Q3	611/1026	6.43 (5.53, 7.46)	2.73 (2.07, 3.61)	2.68 (2.02, 3.55)
Q4	733/1026	13.41 (11.25, 15.98)	5.05 (3.66, 6.98)	4.71 (3.43, 6.46)
*P*_trend_		< 0.001	< 0.001	< 0.001
CVD mortality				
Per 10 years increase	782/4104	1.96 (1.84, 2.10)	1.62 (1.48, 1.78)	1.63 (1.45, 1.84)
By quartile				
Q1	62/1026	1.00 (reference)	1.00 (reference)	1.00 (reference)
Q2	155/1026	3.89 (2.78, 5.43)	1.97 (1.33, 2.91)	1.86 (1.23, 2.81)
Q3	262/1026	8.50 (6.09, 11.86)	3.12 (1.97, 4.96)	2.98 (1.77, 5.01)
Q4	303/1026	17.27 (12.23, 24.39)	5.58 (3.44, 9.08)	5.10 (3.00, 8.68)
*P*_trend_		< 0.001	< 0.001	< 0.001
Non‐CVD mortality				
Per 10 years increase	1137/4104	1.85 (1.75, 1.97)	1.66 (1.49, 1.85)	1.71 (1.54, 1.91)
By quartile				
Q1	129/1026	1.00 (reference)	1.00 (reference)	1.00 (reference)
Q2	229/1026	2.75 (2.09, 3.62)	1.65 (1.18, 2.31)	1.60 (1.15, 2.22)
Q3	349/1026	5.43 (4.38, 6.74)	2.54 (1.79, 3.59)	2.52 (1.76, 3.61)
Q4	430/1026	11.51 (9.23, 14.36)	4.79 (3.25, 7.06)	4.49 (3.05, 6.60)
*P*_trend_		< 0.001	< 0.001	< 0.001
PhenoAge acceleration				
All‐cause mortality				
Per 5 years increase	1919/4104	1.30 (1.25, 1.36)	1.28 (1.23, 1.34)	1.30 (1.24, 1.36)
By quartile				
Q1	425/1026	1.00 (reference)	1.00 (reference)	1.00 (reference)
Q2	449/1026	1.30 (1.08, 1.56)	1.30 (1.10, 1.53)	1.29 (1.08, 1.54)
Q3	490/1026	1.89 (1.58, 2.26)	1.67 (1.43, 1.94)	1.65 (1.39, 1.95)
Q4	555/1026	3.27 (2.68, 3.98)	3.02 (2.49, 3.66)	3.11 (2.52, 3.83)
*P*_trend_		< 0.001	< 0.001	< 0.001
By binary				
Biologically younger	770/1828	1.00 (reference)	1.00 (reference)	1.00 (reference)
Biologically older	1140/2276	1.99 (1.76, 2.25)	1.86 (1.68, 2.05)	1.81 (1.64, 2.00)
CVD mortality				
Per 5 years increase	782/4104	1.29 (1.23, 1.35)	1.27 (1.22, 1.33)	1.28 (1.20, 1.36)
By quartile				
Q1	180/1026	1.00 (reference)	1.00 (reference)	1.00 (reference)
Q2	180/1026	1.18 (0.91, 1.51)	1.17 (0.93, 1.46)	1.08 (0.85, 1.36)
Q3	202/1026	1.84 (1.49, 2.26)	1.59 (1.31, 1.92)	1.51 (1.22, 1.86)
Q4	220/1026	3.07 (2.40, 3.91)	2.80 (2.26, 3.48)	2.70 (2.09, 3.50)
*P*_trend_		< 0.001	< 0.001	< 0.001
By binary				
Biologically younger	324/1828	1.00 (reference)	1.00 (reference)	1.00 (reference)
Biologically older	458/2276	1.94 (1.65, 2.27)	1.79 (1.55, 2.06)	1.73 (1.49, 2.02)
Non‐CVD mortality				
Per 5 years increase	1137/4104	1.31 (1.25, 1.37)	1.29 (1.22, 1.36)	1.31 (1.24, 1.38)
By quartile				
Q1	245/1026	1.00 (reference)	1.00 (reference)	1.00 (reference)
Q2	269/1026	1.38 (1.09, 1.74)	1.39 (1.10, 1.75)	1.45 (1.15, 1.84)
Q3	288/1026	1.93 (1.53, 2.43)	1.72 (1.38, 2.13)	1.75 (1.39, 2.19)
Q4	335/1026	3.41 (2.71, 4.28)	3.16 (2.49, 4.03)	3.40 (2.63, 4.40)
*P*_trend_		< 0.001	< 0.001	< 0.001
By binary				
Biologically younger	455/1828	1.00 (reference)	1.00 (reference)	1.00 (reference)
Biologically older	682/2276	2.02 (1.75, 2.34)	1.90 (1.67, 2.17)	1.86 (1.62, 2.13)

*Note:* Model 1 was crude model; model 2 was adjusted for age, gender, smoking, alcohol consumption, education and family PIR; model 3 was additionally adjusted for BMI, hypertension, diabetes, dyslipidemia, and CHD.

Abbreviations: BMI, body mass index; CHD, coronary heart disease; CVD, cardiovascular disease; HR, hazard ratio; PhenoAge, phenotypic age; PIR, poverty‐to‐income ratio; 95% CI, 95% confidence interval.

Time‐dependent ROC analyses showed that PhenoAge acceleration maintained robust and stable predictive efficacy for all‐cause, CVD‐specific, and non‐CVD mortality, with the highest discrimination observed for 10‐year outcomes ranging steadily from 0.809 to 0.815 (Figure S3).

### 3.4 Mediating Roles of AD and PD in Biological Aging‐Related Mortality Risk

**FIGURE 2:**
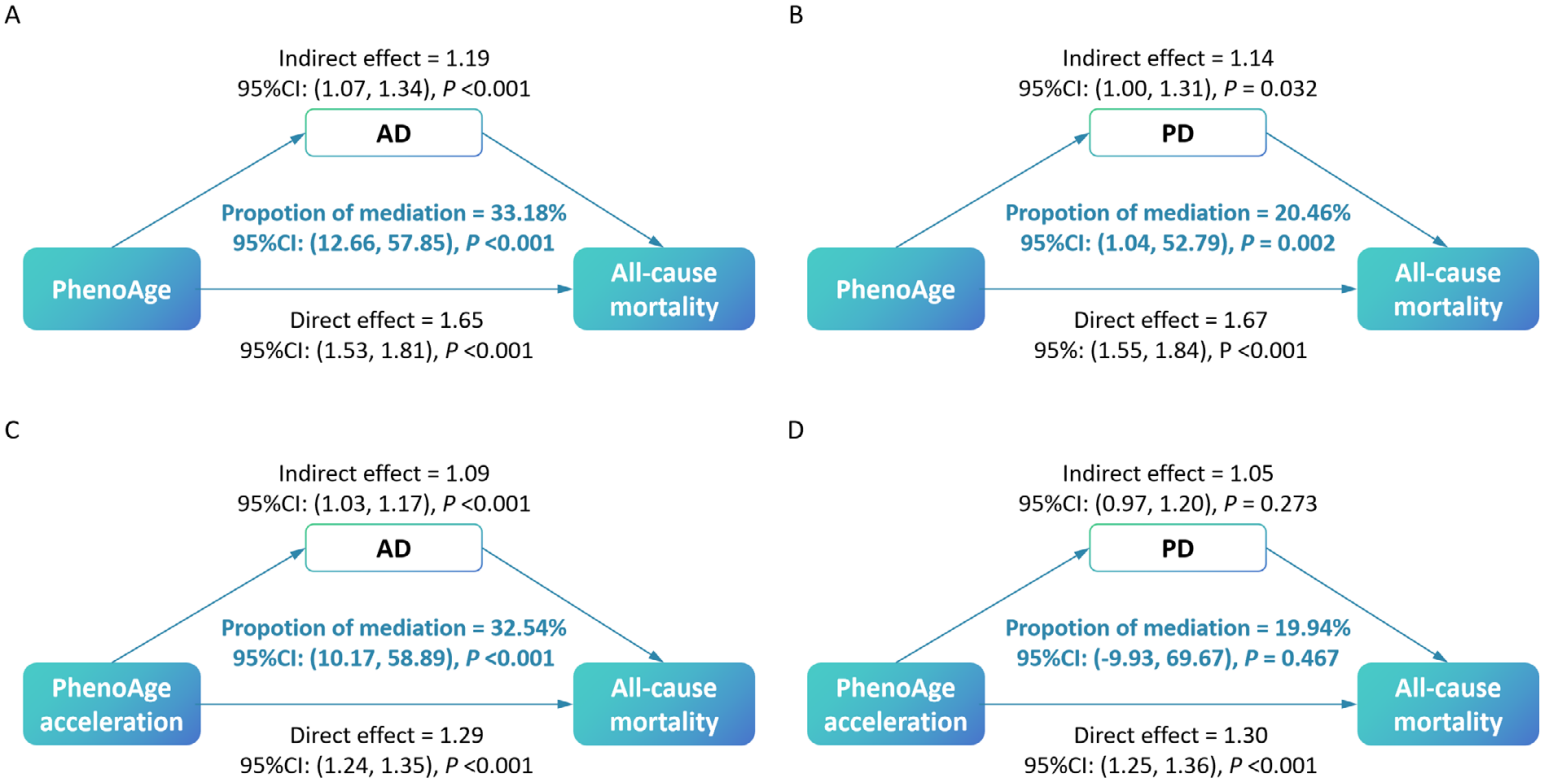
Mediation effects of AD and PD on associations of PhenoAge and acceleration with all‐cause mortality risk among CVD patients. Abbreviations: AD, Alzheimer's disease; BMI, body mass index; CHD, coronary heart disease; CVD, cardiovascular disease; PD, Parkinson's disease; PhenoAge, phenotypic age; PIR, poverty‐to‐income ratio; 95% CI, 95% CI confidence interval. Models were adjusted for age, gender, smoking, alcohol consumption, education, family PIR, BMI, hypertension, diabetes, dyslipidemia, and CHD.

Mediation analysis revealed that AD and PD partially mediated the association between biological aging and mortality (Figure 2). AD significantly mediated 33.18% (*p* < 0.001) (indirect effect = 1.19; 95% CI: 1.07, 1.34) and 32.54% (*p* < 0.001) (indirect effect = 1.09; 95% CI: 1.03, 1.17) of the associations of PhenoAge and PhenoAge acceleration with all‐cause mortality, respectively. PD mediated 20.46% (*p* = 0.002) (indirect effect = 1.14; 95% CI: 1.00, 1.31) of the association between PhenoAge and all‐cause mortality, while its mediating effect on PhenoAge acceleration‐associated mortality did not achieve statistical significance (*p* = 0.467) (indirect effect = 1.05; 95% CI: 0.97, 1.20). Stratified cause‐specific mortality analyses showed that AD stably mediated the links between PhenoAge and acceleration with both CVD and non‐CVD mortality, while PD only participated in PhenoAge‐associated non‐CVD mortality pathways (Figure S6).

### 3.5 Modification Effects of CVD Onset Age and Lifestyle

When stratified by CVD onset age, early‐onset CVD (< 55 years) markedly amplified the detrimental impacts of biological aging on long‐term survival prognosis (Figure S7). The HRs (95% CIs) of PhenoAge and acceleration for all‐cause mortality were 1.46 (1.32, 1.63) and 1.21 (1.09, 1.36) in early‐onset patients and 1.22 (1.16, 1.29) and 1.12 (1.05, 1.19) in late‐onset individuals, with significant multiplicative interaction (*P*_interaction_ < 0.05). For adverse brain health outcomes, cognitive dysfunction risk associated with PhenoAge was elevated in the early‐onset subgroup with borderline interactive significance (*P*_interaction_ = 0.071), while no prominent modification effect was observed for AD and PD prevalence.

In analyses stratified by lifestyle score, healthy lifestyle patterns partially mitigated CVD mortality risks associated with PhenoAge and acceleration (Figure S8). Compared to patients with higher lifestyle scores, those with lower lifestyle scores exhibited much stronger associations of PhenoAge (HR = 1.75 vs. 1.57, *P*_intercation_ = 0.049) and PhenoAge acceleration (HR = 1.33 vs. 1.24, *P*_intercation_ = 0.069) with CVD mortality. The marginally significant interaction needs cautious interpretation and warrants verification in larger samples. No statistically significant modification of lifestyle was detected for other associations.

Other subgroup analyses stratified by hypertension, diabetes, and dyslipidemia found that the association of PhenoAge with cognitive dysfunction was significantly stronger in participants with dyslipidemia, and the impact of PhenoAge acceleration on CVD mortality was greater in individuals without diabetes (Table S4). The results of the sensitivity analyses were robust (Tables S5 and S6 and Figure S9).

## 4 Discussion

In this large, population‐based longitudinal cohort of patients with CVD, we demonstrated that PhenoAge and acceleration were robustly and independently associated with elevated risks of NDD and mortality, while also showing favorable predictive performance for these endpoints. NDD, especially AD, partially mediated the association between biological aging and mortality. Moreover, early‐onset CVD markedly amplified the harmful impact of biological aging on survival, and a healthier lifestyle partially alleviated the elevated CVD mortality risk linked to biological aging. Collectively, these findings established PhenoAge as a clinical biomarker for the heart‐brain aging axis, and provide insights into aging‐driven brain injury and premature death among CVD patients.

Aging is a complex biological process with marked individual heterogeneity in its progression, driven by dynamic interactions across hallmark traits over the lifespan. This heterogeneity renders chronological age inherently limited in predicting mortality and adverse health outcomes [25]. In this context, the biological aging concept was proposed as a better indicator to assess lifespan and aging [26]. Previous population‐based studies have established that biological aging markers are associated with elevated risks of all‐cause mortality and adverse brain health outcomes including cognitive decline, AD, and PD [27]. However, most prior investigations were conducted in general community‐dwelling populations and rarely focused on high‐risk individuals with established CVD, a group characterized by accelerated systemic aging and disproportionately high rates of neurodegeneration and premature death [28, 29]. Our study extended these findings by demonstrating that accelerated biological aging was independently and robustly associated with impaired cognitive function, higher risks of prevalent AD and PD, as well as increased long‐term mortality in patients with CVD. ROC analysis indicated that accelerated biological aging had good predictive performance for AD and PD. Notably, it could not only predict all‐cause, CVD, and non‐CVD mortality within a few years (1, 3, and 5 years), but also exhibit a long‐term predictive capacity for 10 years [30]. By integrating neurocognitive outcomes, NDDs, and mortality within a unified cardio‐cerebral aging framework, our findings filled critical knowledge gaps regarding the shared aging etiology of cardio‐cerebral comorbidity, and highlighted biological aging as a promising prognostic biomarker for risk stratification and targeted intervention in this high‐risk population.

Our mediation analysis further revealed a significant cascade whereby biological aging could contribute to excess mortality partially through AD and PD prevalences, which supported a potential mechanistic pathway along the heart‐brain aging axis. This hierarchical pathway implies that neurodegenerative processes may serve as key intermediate links translating systemic physiological dysregulation into adverse long‐term outcomes, rather than mortality being driven directly by cardiovascular aging alone. Plausible mechanisms underlying this heart‐brain aging axis include chronic low‐grade systemic inflammation, impaired cerebral perfusion secondary to vascular dysfunction, blood–brain barrier disruption, and progressive neuronal atrophy, all of which may be accelerated by premature biological aging [6, 31]. Specifically, accelerated biological aging may trigger sustained neuroinflammation and reduce cerebrovascular reserve, thereby promoting amyloid‐beta aggregation and dopaminergic neuronal loss characteristic of AD and PD pathogenesis [2, 32]. These pathophysiological changes may further compromise central nervous system integrity and accelerate multi‐organ deterioration, ultimately increasing mortality risk. Taken together, these results underscore the integral role of neurodegeneration in mediating the adverse prognostic impact of accelerated biological aging, and provide novel mechanistic insights into the shared biological underpinnings of CVD, neurodegeneration, and premature death. Nevertheless, it is important to note that in our study, NDDs were defined based on prevalent cases at baseline rather than incident cases during follow‐up, so the evidence from our mediation analysis is relatively limited. However, PhenoAge, as a composite measure derived from blood biomarkers, can capture long‐term cumulative physiological damage, and clinical diagnoses of AD and PD typically occur at a relatively late stage of disease, after years of subclinical neurodegeneration. Therefore, prevalent NDDs at baseline might be reasonably viewed as a downstream consequence of prior accelerated biological aging. Future studies with longitudinal assessments of NDDs are needed to confirm our findings.

Meanwhile, the stronger positive associations among early‐onset CVD patients with significant interactions suggested that they were more sensitive to biological aging acceleration. These trends were similar to previous studies on onset age of some chronic diseases [33, 34]. Individuals with early‐onset CVD may experience a longer period of pathological alterations, metabolic dysregulation, and chronic inflammation [35, 36], which might lead to accelerated biological aging and result in higher mortality risks compared to those with late‐onset CVD. Another possible explanation is that early‐onset CVD may be partly attributable to genetic predisposition. Several studies have found a considerable number of genetic variants that are most likely associated with early‐onset CVD, such as lipid and blood pressure‐related single‐nucleotide polymorphisms [37, 38]. Patients with early‐onset CVD might carry CVD‐related genetic variants and experience a greater cumulative cardiovascular and overall health burden throughout their lives, resulting in the markedly higher excess mortality [39, 40]. Therefore, the elevated mortality risk among patients with early‐onset CVD warrants sustained and specialized attention. For these younger patients, biological aging assessment can be considered a valuable adjunct to routine clinical practice and follow‐up care, enabling timely intervention when accelerated biological aging is detected. Lifestyle intervention represents a readily accessible approach at the individual level, so we analyzed the potential beneficial effect of lifestyle in the associations between biological aging and mortality [41]. Results showed that a healthier lifestyle could partially attenuate the positive associations between PhenoAge and CVD mortality with significant interaction, suggesting that lifestyle could be a feasible intervention to reduce aging‐related risks for CVD patients. Given the small sample size in lifestyle‐stratified analyses and insufficient statistical power, the effects of lifestyle intervention should be interpreted cautiously.

Based on a US nationally representative longitudinal survey, this study innovatively explored the role of systemic biological aging in the heart–brain connection and subsequent prognosis, and analyzed the modification effects of CVD onset age and lifestyles, providing novel targets for risk stratification and intervention. Some limitations should be acknowledged. First, related covariates were collected using self‐reported questionnaires, which might introduce recall bias and affect information accuracy. Second, clinical biomarkers were measured from a single blood collection and might not accurately evaluate participants' usual levels due to the dynamic nature of these physiological measures. Third, mediation analysis did not strictly satisfy the temporal sequence, requiring further validation. Fourth, the ascertainment of AD and PD based on self‐reported use of prescribed medication might lead to limited sensitivity and conservative estimation, and accurate and reliable diagnostic criteria should be applied in future studies. Finally, this investigation was conducted among US participants and the generalizability to other ethnic populations requires further verification.

## 5 Conclusion

This study indicated that biological aging was a robust and independent prognostic factor in CVD patients. Its detrimental effects on long‐term outcomes were partially mediated by neurodegenerative processes. Early‐onset CVD and healthier lifestyle had modification effects on these harmful associations. These findings emphasized biological aging as a potential modifiable target for risk stratification and preventive interventions to improve the long‐term prognosis of this high‐risk population.

## Author Contributions

Li Jinyue contributed to conceptualization, methodology, formal analysis, and writing – original draft; Ma Han contributed to data curation, validation, and writing – review and editing; Wang Guohua contributed to supervision, project administration, and writing – review and editing. The final version of the manuscript has been read and approved by all authors.

## Funding

This research was supported by the Capital's Funds for Health lmprovement and Research (CFH) (Grant: 2026‐2G‐20111); the Scientific Research Project of Xuanwu Hospital, Capital Medical University (Grant: QNPY202518).

## Disclosure

The authors have nothing to report.

## Ethics Statement

The NHANES study protocols were approved by the National Center for Health Statistics (NCHS) Research Ethics Review Board (ERB). All participants provided written informed consent prior to participation in NHANES. Our study utilized only publicly available, de‐identified data from NHANES.

## Conflicts of Interest

The authors declare no conflicts of interest.

## Supporting information


**Figure S1:** Flowchart for inclusion and exclusion of study participants.
**Figure S2:** The exposure‐response curves of the associations of PhenoAge and acceleration with AD and PD risks among CVD patients.
**Figure S3:** ROC for the prediction of PhenoAge acceleration for cognitive dysfunction, AD, PD, and mortality risks among CVD patients.
**Figure S4:** Kaplan–Meier survival curves for all‐cause, CVD, and non‐CVD mortality risks with PhenoAge and acceleration among CVD patients.
**Figure S5:** The exposure‐response curves of the associations of PhenoAge and acceleration with all‐cause, CVD, and non‐CVD mortality risks among CVD patients.
**Figure S6:** Mediation effects of AD and PD on associatons of PhenoAge and acceleration with CVD and non‐CVD mortality risk among CVD patients.
**Figure S7:** Associations of PhenoAge and acceleration with cognitive dysfunction, AD, PD, and mortality risks among early‐onset and late‐onset CVD patients.
**Figure S8:** Associations of PhenoAge and acceleration with cognitive dysfunction, AD, PD, and mortality risks among CVD patients stratified by lifestyle score.
**Figure S9:** ROC for the prediction of PhenoAge acceleration for cognitive dysfunction, AD, PD and mortality risks in the sensitivity analyses excluding patients with cancer and kidney failure.
**Table S1:** Descriptive characteristics of study population by PhenoAge acceleration quartiles among patients who underwent cognitive function assessment.
**Table S2:** Associations of PhenoAge and acceleration with AD and mortality risks among stroke patients.
**Table S3:** Associations of PhenoAge and acceleration with AD and mortality risks among coronary heart disease patients.
**Table S4:** Associations of PhenoAge and acceleration with cognitive dysfunction, AD, PD and mortality risks among CVD patients stratified by hypertension, diabetes and dyslipidemia status.
**Table S5:** Associations of PhenoAge and acceleration with neurodegenerative diseases and mortality risks in the sensitivity analyses.
**Table S6:** Mediation effects of AD and PD on associations of PhenoAge and acceleration with all‐cause mortality risk among CVD patients in the sensitivity analyses.

## Data Availability

The data that support the findings of this study are openly available in NHANES at https://www.cdc.gov/nchs/nhanes/.
