# Supplementary material for: Accelerated Biological Aging, Neurodegenerative Disease, and Mortality in Cardiovascular Disease Patients: Mediation and Modification Analysis
Source: CNS Neurosci Ther. 2026 Jul 29;32(8):e71059. doi: 10.1002/cns.71059 (PMC13418375; doi:10.1002/cns.71059)
Supplement: Supplementary file 1 — Figure S1: Flowchart for inclusion and exclusion of study participants. Figure S2: The exposure‐response curves of the associations of PhenoAge and acceleration with AD and PD risks among CVD patients. Figure S3: ROC for the prediction of PhenoAge acceleration for cognitive dysfunction, AD, PD, and mortality risks among CVD patients. Figure S4: Kaplan–Meier survival curves for all‐cause, CVD, and non‐CVD mortality risks with PhenoAge and acceleration among CVD patients. Figure S5: The exposure‐response curves of the associations of PhenoAge and acceleration with all‐cause, CVD, and non‐CVD mortality risks among CVD patients. Figure S6: Mediation effects of AD and PD on associatons of PhenoAge and acceleration with CVD and non‐CVD mortality risk among CVD patients. Figure S7: Associations of PhenoAge and acceleration with cognitive dysfunction, AD, PD, and mortality risks among early‐onset and late‐onset CVD patients. Figure S8: Associations of PhenoAge and acceleration with cognitive dysfunction, AD, PD, and mortality risks among CVD patients stratified by lifestyle score. Figure S9: ROC for the prediction of PhenoAge acceleration for cognitive dysfunction, AD, PD and mortality risks in the sensitivity analyses excluding patients with cancer and kidney failure. Table S1: Descriptive characteristics of study population by PhenoAge acceleration quartiles among patients who underwent cognitive function assessment. Table S2: Associations of PhenoAge and acceleration with AD and mortality risks among stroke patients. Table S3: Associations of PhenoAge and acceleration with AD and mortality risks among coronary heart disease patients. Table S4: Associations of PhenoAge and acceleration with cognitive dysfunction, AD, PD and mortality risks among CVD patients stratified by hypertension, diabetes and dyslipidemia status. Table S5: Associations of PhenoAge and acceleration with neurodegenerative diseases and mortality risks in the sensitivity analyses. Table S6: Mediation ef [file CNS-32-e71059-s001.docx]

**Supplementary Materials**

Accelerated Biological Aging, Neurodegenerative Disease, and Mortality in Cardiovascular Disease Patients: Mediation and Modification Analysis

Jinyue Li ^1,2^, Han Ma ^3 *^, Guohua Wang ^1,2 *^

^1^ National Stroke Administration Office, Xuanwu Hospital Capital Medical University, Bejing, China

^2^ Stroke Prevention, Treatment & Translational Research Laboratory, Xuanwu Hospital Capital Medical University, Beijing, China

^3^ National Center for Clinical Laboratories, Beijing Hospital, National Center for Gerontology, National Clinical Research Center for Gerontology, The Key Laboratory of Geriatrics of NHC, Institute of Geriatric Medicine, Chinese Academy of Medical Sciences, Beijing, China

^*^ Corresponding authors: Han Ma and Guohua Wang

Table S1 Descriptive characteristics of study population by PhenoAge acceleration quartiles among patients who underwent cognitive function assessment

|  | **Total (n=543)** | **PhenoAge acceleration** | | | | ***P*** |
| --- | --- | --- | --- | --- | --- | --- |
|  |  | **Q1 (n=136)** | **Q2 (n=135)** | **Q3 (n=136)** | **Q4 (n=136)** |  |
| Age, years | 73.3±7.8 | 72.5±7.2 | 73.3±8.4 | 74.0±7.8 | 73.4±7.8 | 0.450 |
| Men, n (%) | 322 (59.3) | 63 (46.3) | 80 (59.3) | 90 (66.2) | 89 (65.4) | 0.003 |
| Race, n (%) |  |  |  |  |  | 0.121 |
| Mexican American | 91 (16.8) | 29 (21.3) | 23 (17.0) | 18 (13.2) | 21 (15.4) |  |
| Other Hispanic | 13 (2.4) | 5 (3.7) | 6 (4.4) | 2 (1.5) | 0 (0.0) |  |
| Non-Hispanic White | 366 (67.4) | 86 (63.2) | 95 (70.4) | 94 (69.1) | 91 (66.9) |  |
| Non-Hispanic Black | 66 (12.2) | 14 (10.3) | 10 (7.4) | 19 (14.0) | 23 (16.9) |  |
| Other Race | 7 (1.3) | 2 (1.5) | 1 (0.7) | 3 (2.2) | 1 (0.7) |  |
| Education, n (%) |  |  |  |  |  | 0.761 |
| Less Than 9th Grade | 130 (23.9) | 29 (21.3) | 37 (27.4) | 34 (25.0) | 30 (22.1) |  |
| 9-11th Grade | 95 (17.5) | 21 (15.4) | 22 (16.3) | 24 (17.6) | 28 (20.6) |  |
| High School Grad/GED or Equivalent | 137 (25.2) | 43 (31.6) | 33 (24.4) | 27 (19.9) | 34 (25.0) |  |
| Some College or AA degree | 110 (20.3) | 25 (18.4) | 27 (20.0) | 34 (25.0) | 24 (17.6) |  |
| College Graduate or above | 69 (12.7) | 18 (13.2) | 16 (11.9) | 16 (11.8) | 19 (14.0) |  |
| Family PIR <1, n (%) | 77 (14.2) | 15 (11.0) | 22 (16.3) | 22 (16.2) | 18 (13.2) | 0.542 |
| BMI, kg/m^2^ | 28.1±5.1 | 27.5±4.3 | 27.4±5.3 | 28.2±5.0 | 29.4±5.6 | 0.003 |
| PhenoAge, years | 73.1±10.9 | 64.9±7.8 | 70.0±8.5 | 74.6±8.2 | 83.0±9.8 | <0.001 |
| PhenoAge acceleration, years | -0.2±7.2 | -7.6±1.8 | -3.3±1.1 | 0.6±1.4 | 9.7±6.0 | <0.001 |
| Hypertension, n (%) | 412 (75.9) | 103 (75.7) | 98 (72.6) | 101 (74.3) | 110 (80.9) | 0.415 |
| Dyslipidemia, n (%) | 337 (62.1) | 96 (70.6) | 85 (63.0) | 74 (54.4) | 82 (60.3) | 0.050 |
| DSST score | 37.4±16.3 | 41.1±15.9 | 37.5±17.9 | 36.2±16.5 | 34.6±13.8 | 0.008 |
| Cognitive dysfunction, n (%) | 230 (42.4) | 46 (33.8) | 54 (40.0) | 60 (44.1) | 70 (51.5) | 0.027 |

Abbreviations: BMI, body mass index; CVD, cardiovascular disease; DSST, Digit Symbol Substitution Test; PhenoAge, phenotypic age; PIR, poverty-to-income ratio.

Table S2 Associations of PhenoAge and acceleration with AD and mortality risks among stroke patients

|  | **AD** | | **All-cause mortality** | | **CVD mortality** | | **Non-CVD mortality** | |
| --- | --- | --- | --- | --- | --- | --- | --- | --- |
|  | **Event/N** | **OR (95%CI)** | **Event/N** | **HR (95%CI)** | **Event/N** | **HR (95%CI)** | **Event/N** | **HR (95%CI)** |
| **PhenoAge** |  |  |  |  |  |  |  |  |
| Per 10 years increase | 139/1,367 | 1.59 (1.24, 2.04) | 668/1,367 | 1.66 (1.48, 1.87) | 270/1,367 | 1.56 (1.27, 1.93) | 398/1,367 | 1.71 (1.47, 1.99) |
| By quartile |  |  |  |  |  |  |  |  |
| Q1 | 22/336 | 1.00 (reference) | 52/336 | 1.00 (reference) | 18/336 | 1.00 (reference) | 34/336 | 1.00 (reference) |
| Q2 | 25/341 | 1.16 (0.50, 2.68) | 148/341 | 2.34 (1.40, 3.91) | 57/341 | 3.45 (1.35, 8.80) | 91/341 | 1.92 (1.11, 3.31) |
| Q3 | 35/328 | 1.47 (0.45, 4.78) | 201/328 | 3.08 (1.74, 5.45) | 91/328 | 4.73 (1.74, 12.86) | 110/328 | 2.48 (1.28, 4.83) |
| Q4 | 57/362 | 2.29 (0.72, 7.32) | 267/362 | 5.32 (2.87, 9.84) | 104/362 | 7.25 (2.40, 21.92) | 163/362 | 4.52 (2.33, 8.76) |
| *P*_trend_ |  | 0.427 |  | <0.001 |  | <0.001 |  | <0.001 |
| **PhenoAge acceleration** | | | | | | |  |  |
| Per 5 years increase | 139/1,367 | 1.26 (1.12, 1.43) | 668/1,367 | 1.29 (1.22, 1.37) | 270/1,367 | 1.25 (1.13, 1.39) | 398/1,367 | 1.31 (1.21, 1.41) |
| By quartile |  |  |  |  |  |  |  |  |
| Q1 | 20/335 | 1.00 (reference) | 150/335 | 1.00 (reference) | 65/335 | 1.00 (reference) | 85/335 | 1.00 (reference) |
| Q2 | 26/325 | 1.15 (0.49, 2.71) | 140/325 | 1.53 (1.16, 2.02) | 64/325 | 1.41 (0.94, 2.12) | 76/325 | 1.61 (1.09, 2.37) |
| Q3 | 36/356 | 1.45 (0.64, 3.29) | 175/356 | 1.57 (1.17, 2.11) | 75/356 | 1.51 (0.99, 2.32) | 100/356 | 1.61 (1.12, 2.33) |
| Q4 | 57/351 | 2.82 (1.39, 5.76) | 203/351 | 3.23 (2.48, 4.21) | 66/351 | 2.69 (1.70, 4.26) | 137/351 | 3.54 (2.53, 4.95) |
| *P*_trend_ |  | 0.038 |  | <0.001 |  | <0.001 |  | <0.001 |
| By binary |  |  |  |  |  |  |  |  |
| Biologically younger | 40/580 | 1.00 (reference) | 263/580 | 1.00 (reference) | 120/580 | 1.00 (reference) | 143/580 | 1.00 (reference) |
| Biologically older | 99/787 | 1.69 (1.00, 2.85) | 405/787 | 1.55 (1.29, 1.86) | 150/787 | 1.38 (1.02, 1.88) | 255/787 | 1.65 (1.31, 2.09) |

Abbreviations: AD, Alzheimer's disease; BMI, body mass index; CHD, coronary heart disease; CVD, cardiovascular disease; HR, hazard ratio; PhenoAge, phenotypic age; PIR, poverty-to-income ratio; SD, standard deviation; 95%CI, 95% confidence interval.

Models were adjusted for age, gender, smoking, alcohol consumption, education, family PIR, BMI, hypertension, diabetes, dyslipidemia, and CHD.

Table S3 Associations of PhenoAge and acceleration with AD and mortality risks among coronary heart disease patients

|  | **AD** | | **All-cause mortality** | | **CVD mortality** | | **Non-CVD mortality** | |
| --- | --- | --- | --- | --- | --- | --- | --- | --- |
|  | **Event/N** | **OR (95%CI)** | **Event/N** | **HR (95%CI)** | **Event/N** | **HR (95%CI)** | **Event/N** | **HR (95%CI)** |
| **PhenoAge** |  |  |  |  |  |  |  |  |
| Per 10 years increase | 106/1,561 | 1.44 (1.07, 1.93) | 737/1,561 | 1.62 (1.38, 1.90) | 301/1,561 | 1.61 (1.31, 1.98) | 436/1,561 | 1.62 (1.36, 1.94) |
| By quartile |  |  |  |  |  |  |  |  |
| Q1 | 9/319 | 1.00 (reference) | 68/319 | 1.00 (reference) | 24/319 | 1.00 (reference) | 44/319 | 1.00 (reference) |
| Q2 | 17/413 | 1.38 (0.41, 4.60) | 144/413 | 1.60 (1.13, 2.27) | 57/413 | 1.44 (0.82, 2.54) | 87/413 | 1.72 (1.05, 2.81) |
| Q3 | 39/426 | 3.36 (0.93, 12.19) | 248/426 | 2.72 (1.84, 4.01) | 100/426 | 2.17 (1.10, 4.28) | 148/426 | 3.15 (1.78, 5.59) |
| Q4 | 41/403 | 3.60 (0.83, 15.70) | 277/403 | 5.14 (3.25, 8.14) | 120/403 | 4.48 (2.16, 9.30) | 157/403 | 5.59 (2.94, 10.63) |
| *P*_trend_ |  | 0.091 |  | <0.001 |  | <0.001 |  | <0.001 |
| **PhenoAge acceleration** | | | | | | |  |  |
| Per 5 years increase | 106/1,561 | 1.20 (1.04, 1.39) | 737/1,561 | 1.27 (1.17, 1.38) | 301/1,561 | 1.27 (1.15, 1.41) | 436/1,561 | 1.27 (1.16, 1.39) |
| By quartile |  |  |  |  |  |  |  |  |
| Q1 | 19/397 | 1.00 (reference) | 155/397 | 1.00 (reference) | 72/397 | 1.00 (reference) | 83/397 | 1.00 (reference) |
| Q2 | 21/393 | 1.04 (0.43, 2.54) | 186/393 | 1.34 (1.03, 1.74) | 71/393 | 1.01 (0.70, 1.47) | 115/393 | 1.64 (1.12, 2.40) |
| Q3 | 21/375 | 1.01 (0.45, 2.26) | 178/375 | 1.44 (1.11, 1.87) | 69/375 | 1.19 (0.83, 1.71) | 109/375 | 1.65 (1.14, 2.41) |
| Q4 | 45/396 | 2.11 (0.92, 4.83) | 218/396 | 2.93 (2.11, 4.06) | 89/396 | 2.39 (1.59, 3.59) | 129/396 | 3.42 (2.19, 5.33) |
| *P*_trend_ |  | 0.084 |  | <0.001 |  | <0.001 |  | <0.001 |
| By binary |  |  |  |  |  |  |  |  |
| Biologically younger | 36/710 | 1.00 (reference) | 304/710 | 1.00 (reference) | 125/710 | 1.00 (reference) | 179/710 | 1.00 (reference) |
| Biologically older | 70/851 | 1.57 (1.01, 2.46) | 433/851 | 1.66 (1.40, 1.97) | 176/851 | 1.62 (1.26, 2.09) | 257/851 | 1.69 (1.34, 2.12) |

Abbreviations: AD, Alzheimer's disease; BMI, body mass index; CVD, cardiovascular disease; HR, hazard ratio; PhenoAge, phenotypic age; PIR, poverty-to-income ratio; SD, standard deviation; 95%CI, 95% confidence interval.

Models were adjusted for age, gender, smoking, alcohol consumption, education, family PIR, BMI, hypertension, diabetes, and dyslipidemia.

Table S4 Associations of PhenoAge and acceleration with cognitive dysfunction, AD, PD, and mortality risks among CVD patients stratified by hypertension, diabetes, and dyslipidemia status

|  | Event/N | OR (95%CI) | *P*_interaction_ | Event/N | HR (95%CI) | *P*_interaction_ |
| --- | --- | --- | --- | --- | --- | --- |
| **PhenoAge, per 10 years increase** | | | |  |  |  |
| **Cognitive dysfunction** | | | | **All-cause mortality** | | |
| With hypertension | 187/412 | 1.49 (1.05, 2.10) | 0.297 | 1,514/3,150 | 2.22 (1.89, 2.61) | 0.240 |
| Without hypertension | 43/131 | 2.86 (0.86, 9.48) |  | 405/954 | 2.70 (2.10, 3.47) |  |
| With diabetes | 0/0 | - | **-** | 369/974 | 2.03 (1.56, 2.64) | 0.461 |
| Without diabetes | 230/543 | - |  | 1,550/3,130 | 2.44 (2.12, 2.82) |  |
| With dyslipidemia | 132/337 | 2.22 (1.43, 3.44) | 0.040 | 1,133/2,564 | 2.25 (1.88, 2.70) | 0.613 |
| Without dyslipidemia | 98/206 | 1.29 (0.62, 2.67) |  | 786/1,540 | 2.33 (1.92, 2.82) |  |
| **AD** |  |  |  | **CVD mortality** | | |
| With hypertension | 241/3,150 | 1.62 (1.34, 1.97) | 0.492 | 636/3,150 | 2.14 (1.75, 2.62) | 0.800 |
| Without hypertension | 70/954 | 1.32 (0.86, 2.01) |  | 146/954 | 2.88 (1.66, 4.99) |  |
| With diabetes | 132/974 | 1.37 (1.03, 1.83) | 0.100 | 144/974 | 1.66 (1.16, 2.36) | 0.343 |
| Without diabetes | 179/3,130 | 1.38 (1.07, 1.79) |  | 638/3,130 | 2.50 (2.08, 3.00) |  |
| With dyslipidemia | 209/2,564 | 1.41 (1.12, 1.77) | 0.747 | 462/2,564 | 2.09 (1.68, 2.61) | 0.459 |
| Without dyslipidemia | 102/1,540 | 1.35 (0.95, 1.91) |  | 320/1,540 | 2.45 (1.87, 3.22) |  |
| **PD** |  |  |  | **Non-CVD mortality** | | |
| With hypertension | 57/3,150 | 1.35 (0.89, 2.05) | 0.337 | 878/3,150 | 2.27 (1.87, 2.75) | 0.207 |
| Without hypertension | 15/954 | 2.28 (1.32, 3.95) |  | 259/954 | 2.61 (1.93, 3.53) |  |
| With diabetes | 20/974 | 1.79 (1.08, 2.97) | 0.547 | 225/974 | 2.30 (1.72, 3.08) | 0.307 |
| Without diabetes | 52/3,130 | 1.30 (0.84, 2.00) |  | 912/3,130 | 2.40 (2.01, 2.86) |  |
| With dyslipidemia | 45/2,564 | 1.64 (1.08, 2.49) | 0.948 | 671/2,564 | 2.36 (1.91, 2.93) | 0.907 |
| Without dyslipidemia | 27/1,540 | 1.22 (0.77, 1.93) |  | 466/1,540 | 2.24 (1.76, 2.85) |  |
| **PhenoAge acceleration, per 5 years increase** | | | |  |  |  |
| **Cognitive dysfunction** | | | | **All-cause mortality** | | |
| With hypertension | 187/412 | 1.22 (1.03, 1.45) | 0.270 | 1,514/3,150 | 1.52 (1.40, 1.66) | 0.173 |
| Without hypertension | 43/131 | 1.69 (0.93, 3.08) |  | 405/954 | 1.69 (1.48, 1.93) |  |
| With diabetes | 0/0 | - |  | 369/974 | 1.45 (1.26, 1.67) | 0.216 |
| Without diabetes | 230/543 | - |  | 1,550/3,130 | 1.60 (1.49, 1.73) |  |
| With dyslipidemia | 132/337 | 1.49 (1.19, 1.86) | 0.124 | 1,133/2,564 | 1.54 (1.40, 1.69) | 0.430 |
| Without dyslipidemia | 98/206 | 1.13 (0.79, 1.63) |  | 786/1,540 | 1.56 (1.40, 1.73) |  |
| **AD** |  |  |  | **CVD mortality** | | |
| With hypertension | 241/3,150 | 1.27 (1.16, 1.40) | 0.419 | 636/3,150 | 1.50 (1.34, 1.66) | 0.477 |
| Without hypertension | 70/954 | 1.15 (0.93, 1.42) |  | 146/954 | 1.75 (1.31, 2.33) |  |
| With diabetes | 132/974 | 1.17 (1.01, 1.35) | 0.791 | 144/974 | 1.31 (1.08, 1.57) | 0.023 |
| Without diabetes | 179/3,130 | 1.18 (1.03, 1.34) |  | 638/3,130 | 1.62 (1.47, 1.79) |  |
| With dyslipidemia | 209/2,564 | 1.19 (1.06, 1.33) | 0.253 | 462/2,564 | 1.48 (1.31, 1.66) | 0.207 |
| Without dyslipidemia | 102/1,540 | 1.16 (0.97, 1.38) |  | 320/1,540 | 1.61 (1.39, 1.85) |  |
| **PD** |  |  |  | **Non-CVD mortality** | | |
| With hypertension | 57/3,150 | 1.12 (0.94, 1.35) | 0.101 | 878/3,150 | 1.54 (1.39, 1.71) | 0.297 |
| Without hypertension | 15/954 | 1.55 (1.05, 2.29) |  | 259/954 | 1.66 (1.41, 1.95) |  |
| With diabetes | 20/974 | 1.28 (1.01, 1.61) | 0.167 | 225/974 | 1.55 (1.33, 1.81) | 0.872 |
| Without diabetes | 52/3,130 | 1.10 (0.90, 1.34) |  | 912/3,130 | 1.59 (1.45, 1.74) |  |
| With dyslipidemia | 45/2,564 | 1.24 (1.00, 1.55) | 0.402 | 671/2,564 | 1.57 (1.41, 1.76) | 0.958 |
| Without dyslipidemia | 27/1,540 | 1.08 (0.82, 1.42) |  | 466/1,540 | 1.53 (1.35, 1.74) |  |

Abbreviations: AD, Alzheimer's disease; BMI, body mass index; CHD, coronary heart disease; CVD, cardiovascular disease; HR, hazard ratio; OR, odds ratio; PD, Parkinson's disease; PhenoAge, phenotypic age; PIR, poverty-to-income ratio; 95%CI, 95% confidence interval.

Models were adjusted for age, gender, smoking, alcohol consumption, education, family PIR, BMI, hypertension (for diabetes and dyslipidemia subgroups), diabetes (for hypertension and dyslipidemia subgroups), dyslipidemia (for hypertension and diabetes subgroups), and CHD.

Table S5 Associations of PhenoAge and acceleration with neurodegenerative diseases and mortality risks in the sensitivity analyses

|  | **OR (95%CI) or HR (95%CI)** | | | | | | ***P*_trend_** |
| --- | --- | --- | --- | --- | --- | --- | --- |
|  | **Per 10/5 years increase for PhenoAge/PhenoAge acceleration** | **Q1** | **Q2** | **Q3** | **Q4** | |  |
| 1. **Excluding patients with cancer and kidney failure** | | | | | | | |
| **PhenoAge** |  |  |  |  |  | |  |
| AD | 1.44 (1.18, 1.77) | 1.00 (reference) | 1.91 (0.70, 5.22) | 2.57 (0.85, 7.75) | 3.28 (1.02, 10.49) | | 0.001 |
| PD | 1.88 (1.31, 2.69) | 1.00 (reference) | 3.50 (0.79, 15.48) | 8.82 (1.35, 57.58) | 21.66 (3.92, 119.81) | | <0.001 |
| All-cause mortality | 1.57 (1.39, 1.78) | 1.00 (reference) | 1.70 (1.29, 2.24) | 2.14 (1.54, 2.98) | 3.84 (2.60, 5.67) | | <0.001 |
| CVD mortality | 1.67 (1.45, 1.93) | 1.00 (reference) | 2.03 (1.26, 3.27) | 3.40 (1.95, 5.93) | 5.72 (3.11, 10.55) | | <0.001 |
| Non-CVD mortality | 1.51 (1.30, 1.76) | 1.00 (reference) | 1.53 (1.01, 2.32) | 1.58 (1.04, 2.43) | 3.00 (1.82, 4.96) | | <0.001 |
| **PhenoAge acceleration** |  |  |  |  |  | |  |
| AD | 1.20 (1.08, 1.33) | 1.00 (reference) | 1.47 (0.80, 2.69) | 1.84 (1.06, 3.17) | 2.46 (1.35, 4.46) | | <0.001 |
| PD | 1.37 (1.15, 1.64) | 1.00 (reference) | 0.98 (0.26, 3.67) | 3.55 (1.26, 10.02) | 3.87 (1.29, 11.58) | | <0.001 |
| All-cause mortality | 1.25 (1.18, 1.33) | 1.00 (reference) | 1.33 (1.11, 1.59) | 1.56 (1.29, 1.88) | 2.73 (2.16, 3.39) | | <0.001 |
| CVD mortality | 1.29 (1.20, 1.39) | 1.00 (reference) | 1.14 (0.90, 1.45) | 1.46 (1.11, 1.93) | 2.81 (2.12, 3.73) | | <0.001 |
| Non-CVD mortality | 1.23 (1.14, 1.33) | 1.00 (reference) | 1.46 (1.12, 1.91) | 1.62 (1.25, 2.10) | 2.66 (1.97, 3.61) | | <0.001 |
| 1. **Adjusted for** **medication usage** | | | | | | | |
| **PhenoAge** |  |  |  |  |  | |  |
| AD | 1.27 (0.98, 1.64) | 1.00 (reference) | 3.66 (1.29, 10.40) | 6.06 (1.85, 19.80) | 6.38 (1.84, 22.16) | | 0.045 |
| PD | 1.78 (1.28, 2.48) | 1.00 (reference) | 3.07 (0.60, 15.74) | 7.28 (0.89, 59.50) | 17.51 (2.62, 117.11) | | <0.001 |
| All-cause mortality | 1.55 (1.36, 1.76) | 1.00 (reference) | 1.67 (1.27, 2.19) | 2.07 (1.49, 2.88) | 3.60 (2.42, 5.36) | | <0.001 |
| CVD mortality | 1.61 (1.38, 1.88) | 1.00 (reference) | 1.96 (1.24, 3.12) | 3.19 (1.84, 5.53) | 5.10 (2.76, 9.41) | | <0.001 |
| Non-CVD mortality | 1.50 (1.28, 1.77) | 1.00 (reference) | 1.52 (1.00, 2.30) | 1.56 (1.01, 2.40) | 2.91 (1.74, 4.87) | | <0.001 |
| **PhenoAge acceleration** |  |  |  |  |  | |  |
| AD | 1.13 (0.99, 1.28) | 1.00 (reference) | 1.48 (0.71, 3.08) | 2.42 (1.28, 4.57) | 2.45 (1.27, 4.75) | | <0.001 |
| PD | 1.34 (1.09, 1.65) | 1.00 (reference) | 2.16 (0.34, 13.74) | 5.83 (1.23, 27.64) | | 5.96 (1.11, 32.08) | <0.001 |
| All-cause mortality | 1.24 (1.17, 1.33) | 1.00 (reference) | 1.32 (1.12, 1.56) | 1.65 (1.42, 1.93) | | 3.00 (2.46, 3.65) | <0.001 |
| CVD mortality | 1.27 (1.18, 1.37) | 1.00 (reference) | 1.15 (0.91, 1.44) | 1.52 (1.24, 1.86) | | 2.58 (1.99, 3.34) | <0.001 |
| Non-CVD mortality | 1.23 (1.13, 1.33) | 1.00 (reference) | 1.45 (1.15, 1.83) | 1.75 (1.40, 2.18) | | 3.30 (2.59, 4.21) | <0.001 |

Abbreviations: AD, Alzheimer's disease; BMI, body mass index; CHD, coronary heart disease; CVD, cardiovascular disease; HR, hazard ratio; OR, odds ratio; PD, Parkinson's disease; PhenoAge, phenotypic age; PIR, poverty-to-income ratio; 95%CI, 95% confidence interval.

Models were adjusted for age, gender, smoking, alcohol consumption, education, family PIR, BMI, hypertension, diabetes, dyslipidemia, CHD (and medication usage).

Table S6 Mediation effects of AD and PD on associations of PhenoAge and acceleration with all-cause mortality risk among CVD patients in the sensitivity analyses

|  | Direct effect | | Indirect effect | | Mediation effect | |
| --- | --- | --- | --- | --- | --- | --- |
|  | HR (95%CI) | *P* | HR (95%CI) | *P* | Proportion (95%CI) | *P* |
| 1. **Excluding patients with cancer and kidney failure** |  |  |  |  |  |  |
| PhenoAge → AD → All-cause mortality | 1.55 (1.40, 1.76) | <0.001 | 1.17 (1.02, 1.36) | 0.006 | 30.92 (2.37, 66.07) | 0.003 |
| PhenoAge → PD → All-cause mortality | 1.57 (1.42, 1.78) | <0.001 | 1.33 (1.01, 1.74) | 0.007 | 42.00 (7.76, 82.60) | 0.001 |
| PhenoAge acceleration → AD → All-cause mortality | 1.65 (1.43, 1.93) | <0.001 | 1.18 (1.01, 1.37) | 0.001 | 23.20 (3.07, 58.49) | <0.001 |
| PhenoAge acceleration → PD → All-cause mortality | 1.67 (1.45, 1.96) | <0.001 | 1.10 (0.62, 1.82) | 0.339 | 19.14 (-48.97, 60.52) | 0.654 |
| 1. **Adjusted for medication usage** |  |  |  |  |  |  |
| PhenoAge → AD → All-cause mortality | 1.62 (1.49, 1.76) | <0.001 | 1.18 (1.04, 1.34) | <0.001 | 27.34 (1.19, 58.94) | 0.026 |
| PhenoAge → PD → All-cause mortality | 1.63 (1.50, 1.77) | <0.001 | 1.15 (1.03, 1.27) | <0.001 | 23.76 (0.94, 59.20) | 0.017 |
| PhenoAge acceleration → AD → All-cause mortality | 1.68 (1.42, 2.00) | <0.001 | 1.17 (1.00, 1.36) | 0.019 | 21.09 (2.34, 54.94) | 0.004 |
| PhenoAge acceleration → PD → All-cause mortality | 1.67 (1.40, 1.98) | <0.001 | 1.06 (0.94,1.20) | 0.435 | 15.62 (-25.96, 74.01) | 0.397 |

Abbreviations: AD, Alzheimer's disease; BMI, body mass index; CHD, coronary heart disease; CVD, cardiovascular disease; PD, Parkinson's disease; PhenoAge, phenotypic age; PIR, poverty-to-income ratio; 95%CI, 95% confidence interval.

Models were adjusted for age, gender, smoking, alcohol consumption, education, family PIR, BMI, hypertension, diabetes, dyslipidemia, and CHD.


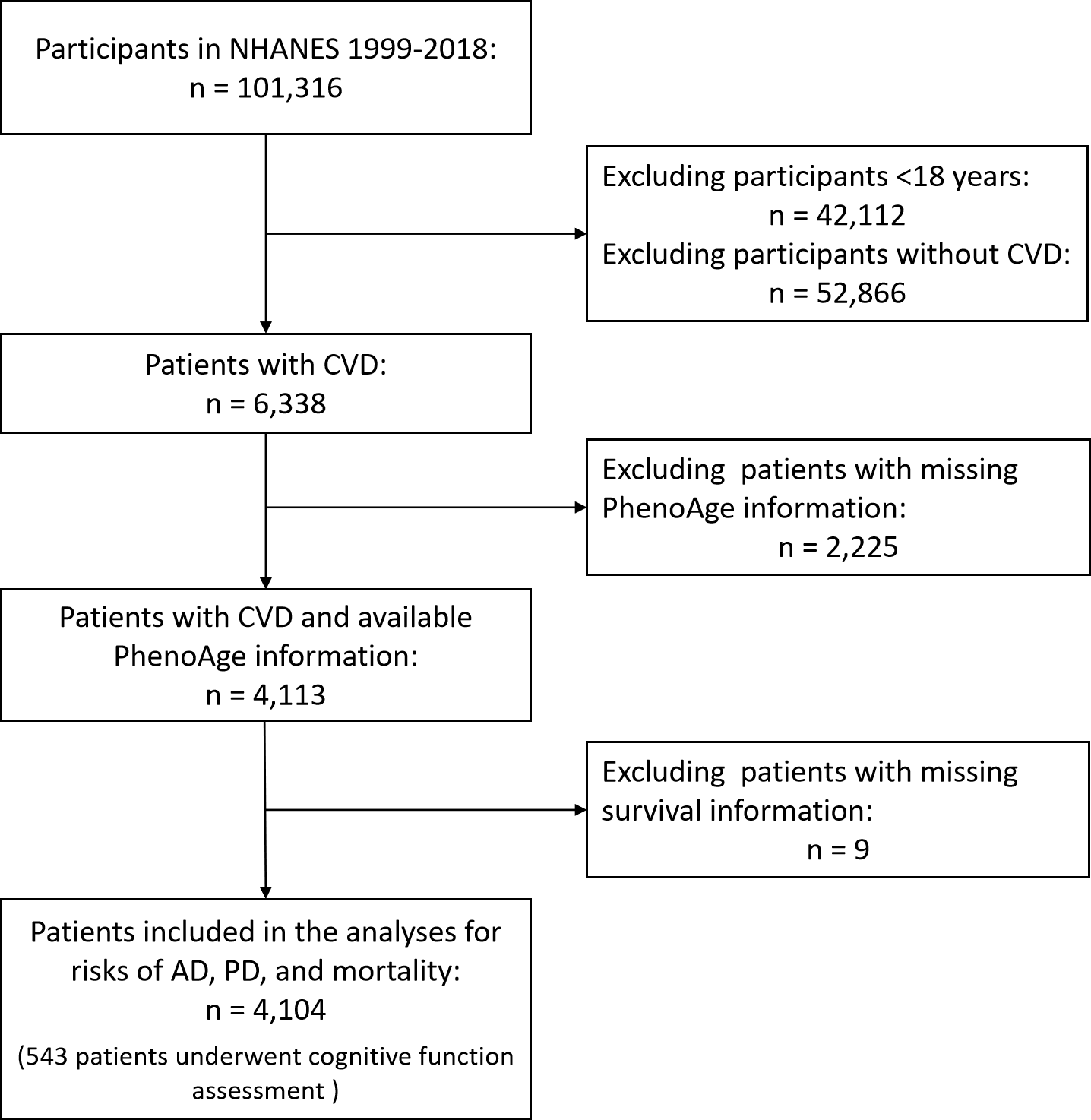


Figure S1 Flowchart for inclusion and exclusion of study participants

Abbreviations: AD, Alzheimer's disease; CVD, cardiovascular disease; NHANES, National Health and Nutrition Examination Survey; PD, Parkinson's disease; PhenoAge, phenotypic age.

Data used in the present analysis were retrieved from the NHANES, and detailed information of the exposure and outcomes can be found in previously published studies [1-5].


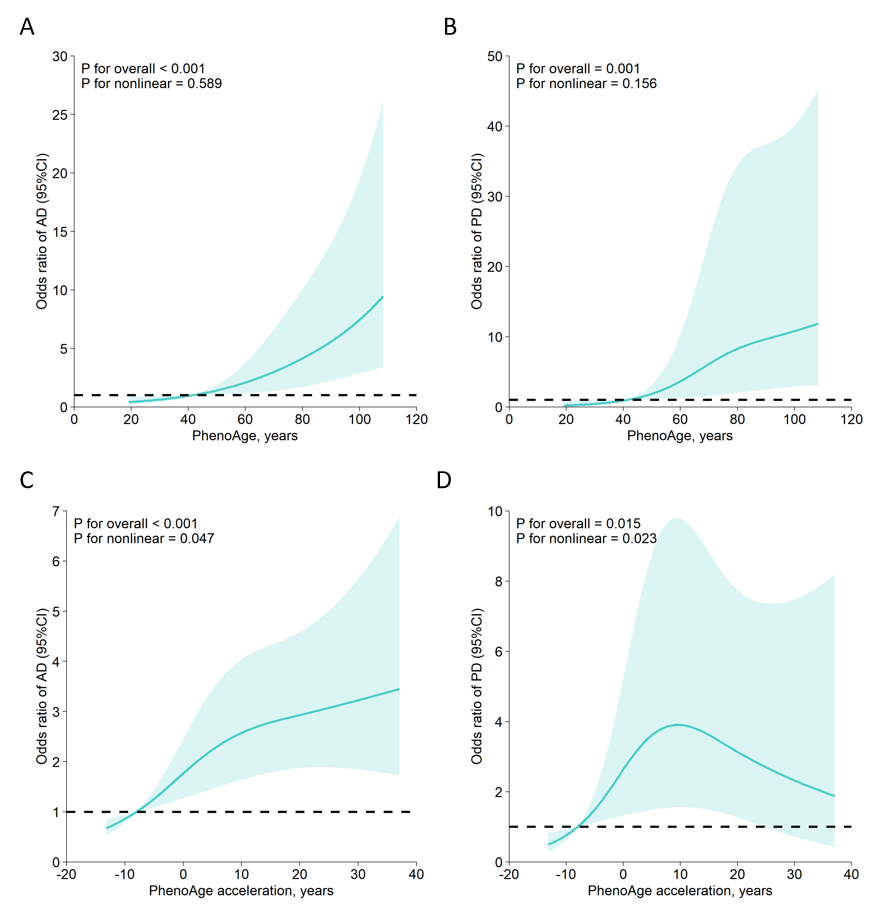


Figure S2 The exposure-response curves of the associations of PhenoAge and acceleration with AD and PD risks among CVD patients

Abbreviations: AD, Alzheimer's disease; BMI, body mass index; CHD, coronary heart disease; CVD, cardiovascular disease; PD, Parkinson's disease; PhenoAge, phenotypic age; PIR, poverty-to-income ratio; 95%CI, 95% confidence interval.

Models were adjusted for age, gender, smoking, alcohol consumption, education, family PIR, BMI, hypertension, diabetes, dyslipidemia, and CHD.

Figure S3 ROC for the prediction of PhenoAge acceleration for cognitive dysfunction, AD, PD, and mortality risks among CVD patients

Abbreviations: AD, Alzheimer's disease; AUC, area under the curve; BMI, body mass index; CHD, coronary heart disease; CVD, cardiovascular disease; PD, Parkinson's disease; PhenoAge, phenotypic age; PIR, poverty-to-income ratio; ROC, receiver operating characteristic.

Figure S4 Kaplan-Meier survival curves for all-cause, CVD, and non-CVD mortality risks with PhenoAge and acceleration among CVD patients

Abbreviations: CVD, cardiovascular disease; PhenoAge, phenotypic age.

Figure S5 The exposure-response curves of the associations of PhenoAge and acceleration with all-cause, CVD, and non-CVD mortality risks among CVD patients

Abbreviations: BMI, body mass index; CHD, coronary heart disease; CVD, cardiovascular disease; PhenoAge, phenotypic age; PIR, poverty-to-income ratio; 95%CI, 95% confidence interval.

Models were adjusted for age, gender, smoking, alcohol consumption, education, family PIR, BMI, hypertension, diabetes, dyslipidemia, and CHD.


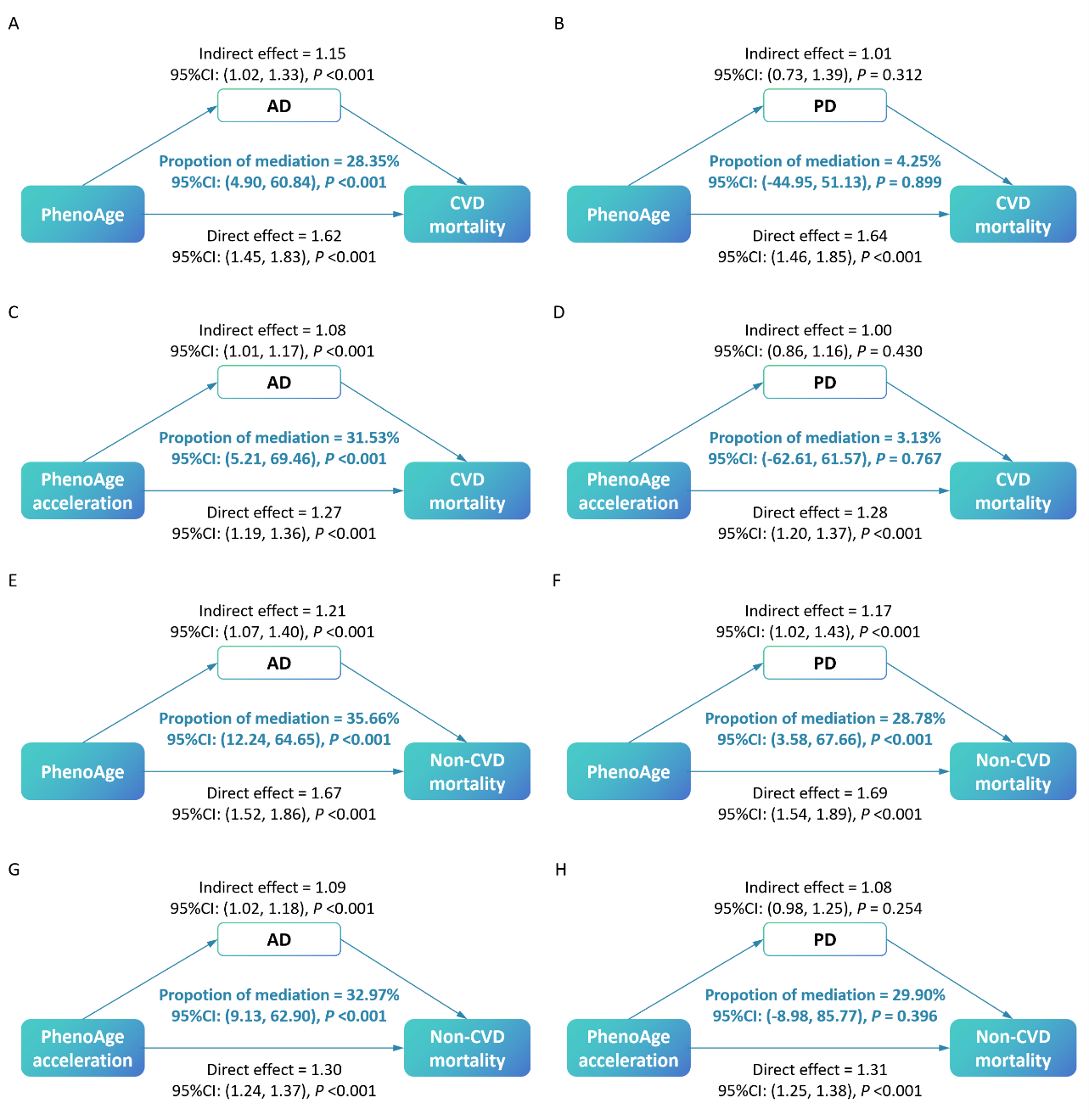


Figure S6 Mediation effects of AD and PD on associations of PhenoAge and acceleration with CVD and non-CVD mortality risk among CVD patients

Abbreviations: AD, Alzheimer's disease; BMI, body mass index; CHD, coronary heart disease; CVD, cardiovascular disease; PD, Parkinson's disease; PhenoAge, phenotypic age; PIR, poverty-to-income ratio; 95%CI, 95% confidence interval.

Models were adjusted for age, gender, smoking, alcohol consumption, education, family PIR, BMI, hypertension, diabetes, dyslipidemia, and CHD.

Figure S7 Associations of PhenoAge and acceleration with cognitive dysfunction, AD, PD, and mortality risks among early-onset and late-onset CVD patients

Abbreviations: AD, Alzheimer's disease; BMI, body mass index; CHD, coronary heart disease; CVD, cardiovascular disease; HR, hazard ratio; OR, odds ratio; PD, Parkinson's disease; PhenoAge, phenotypic age; PIR, poverty-to-income ratio; 95%CI, 95% confidence interval.

Models were adjusted for age, gender, smoking, alcohol consumption, education, family PIR, BMI, hypertension, diabetes, dyslipidemia, and CHD.

Figure S8 Associations of PhenoAge and acceleration with cognitive dysfunction, AD, PD, and mortality risks among CVD patients stratified by lifestyle score

Abbreviations: AD, Alzheimer's disease; BMI, body mass index; CHD, coronary heart disease; CVD, cardiovascular disease; HR, hazard ratio; OR, odds ratio; PD, Parkinson's disease; PhenoAge, phenotypic age; PIR, poverty-to-income ratio; 95%CI, 95% confidence interval.

Models were adjusted for age, gender, education, family PIR, BMI, hypertension, diabetes, dyslipidemia, and CHD.

Figure S9 ROC for the prediction of PhenoAge acceleration for cognitive dysfunction, AD, PD, and mortality risks in the sensitivity analyses excluding patients with cancer and kidney failure

Abbreviations: AD, Alzheimer's disease; AUC, area under the curve; BMI, body mass index; CHD, coronary heart disease; CVD, cardiovascular disease; PD, Parkinson's disease; PhenoAge, phenotypic age; PIR, poverty-to-income ratio; ROC, receiver operating characteristic.

**References**

1. Liu Z, Kuo PL, Horvath S, Crimmins E, Ferrucci L, Levine M: **A new aging measure captures morbidity and mortality risk across diverse subpopulations from NHANES IV: A cohort study**. *PLoS Med* 2018, **15**(12):e1002718.

2. Levine ME, Lu AT, Quach A, Chen BH, Assimes TL, Bandinelli S, Hou L, Baccarelli AA, Stewart JD, Li Y *et al*: **An epigenetic biomarker of aging for lifespan and healthspan**. *Aging (Albany NY)* 2018, **10**(4):573-591.

3. Jaeger J: **Digit Symbol Substitution Test: The Case for Sensitivity Over Specificity in Neuropsychological Testing**. *J Clin Psychopharmacol* 2018, **38**(5):513-519.

4. Dong X, Bai M, Qian J, Xiao J, Zhang S, Hou X, Zhou C: **Harmful impact of blood lead on the prevalence of Alzheimer's disease among middle-aged and older adults and the modifying role of physical activity: Evidence from the National Health and Nutrition Examination Survey (NHANES) study**. *Alzheimers Dement* 2025, **21**(11):e70785.

5. Zhao J, Li F, Wu Q, Cheng Y, Liang G, Wang X, Fang S, Wang Q, Fan X, Fang J: **Association between trichlorophenols and neurodegenerative diseases: A cross-sectional study from NHANES 2003-2010**. *Chemosphere* 2022, **307**(Pt 2):135743.
